# Refractory remimazolam-induced anaphylaxis and suspected Kounis syndrome requiring extracorporeal cardiopulmonary resuscitation: a case report

**DOI:** 10.1186/s40981-026-00862-8

**Published:** 2026-05-26

**Authors:** Hajime Kotsubo, Takumi Taniguchi

**Affiliations:** Department of Anesthesia and Palliative Care, Kanazawa University Hospital, 13-1 Takara-Machi, Kanazawa-City, Ishikawa-Prefecture 920-8641 Japan

**Keywords:** Anaphylaxis, Kounis syndrome, Remimazolam, Extracorporeal cardiopulmonary resuscitation

## Abstract

**Background:**

Kounis syndrome (KS) is an allergic acute coronary syndrome that may precipitate severe perioperative cardiovascular collapse. This study reports a case of remimazolam-induced refractory anaphylaxis complicated by KS and successfully treated with early extracorporeal cardiopulmonary resuscitation (ECPR).

**Case presentation:**

A 69-year-old male with atrial fibrillation was scheduled for lung resection. After induction of anesthesia with remimazolam, he developed abrupt tachycardia, coughing, and circulatory collapse with ST-segment elevation. Despite repeated adrenaline doses, he progressed to cardiac arrest. Venoarterial extracorporeal membrane oxygenation was initiated during cardiopulmonary resuscitation. Return of spontaneous circulation occurred 15 min after the cardiac arrest. The patient recovered without neurological sequelae. Elevated serum tryptase levels (49.5 µg/L; 24-h postonset: 4.5 µg/L) and positive intradermal testing suggested remimazolam-induced anaphylaxis. Coronary computed tomography revealed no stenosis, compatible with suspected type 1 KS.

**Conclusions:**

Remimazolam-induced anaphylaxis may be complicated by suspected KS. In refractory anaphylaxis complicated by cardiac arrest, early ECPR may be critical to survival.

## Background

Anaphylaxis is a potentially lethal complication during anesthesia. In some cases, allergic acute coronary syndrome is complicated and reported as Kounis syndrome (KS). KS is triggered by inflammatory mediators released during mast cell degranulation [1]. It is classified into three types, with type 1 being the most common and marked by coronary artery spasms without major stenosis [2]. Perioperative KS often presents as severe, refractory anaphylaxis. Although management strategies for such cases are not fully established, extracorporeal cardiopulmonary resuscitation (ECPR) should be considered in cardiac arrest that is not responsive to conventional resuscitation [3]. In this case report, we describe a case in which early ECPR was critical for a patient with suspected KS and cardiac arrest after remimazolam administration. Written informed consent was obtained from the patient for the publication of this report.

## Case presentation

A 69-year-old male (height: 169 cm; weight: 78.4 kg) was scheduled for left upper lobe resection. His medical history included persistent atrial fibrillation, hypertension, and diabetes mellitus, managed with rivaroxaban, amlodipine, vildagliptin, and metformin. He had no history of beta-blocker use, drug allergies, or prior general anaesthesia. Notably, he had undergone six gastrointestinal endoscopies under midazolam sedation over 2 years ago. A preoperative electrocardiogram (ECG) showed only atrial fibrillation, and other examinations revealed no abnormalities. Echocardiography showed preserved left ventricular function.

**Fig. 1:**
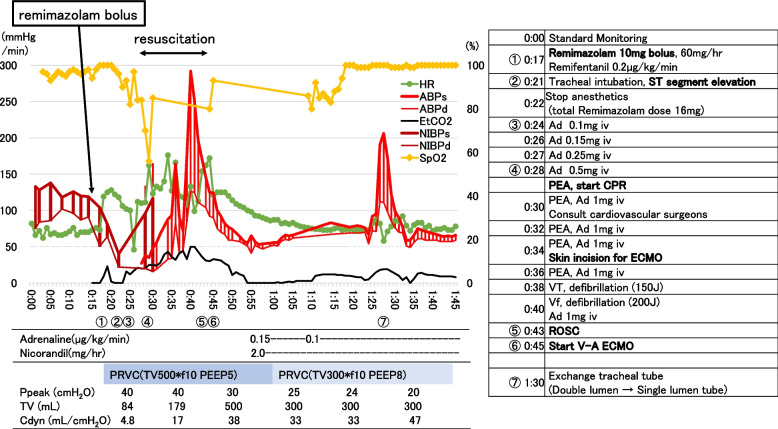
Anesthesia and resuscitation course in operating room. HR; heart rate, ABPs; systolic arterial blood pressure, ABPd; diastolic arterial blood pressure, EtCO2; end-tidal CO2, NIBPs; systolic noninvasive blood pressure, NIBPd; diastolic noninvasive blood, PRVC; pressure regulated volume control, TV; tidal volume, f; respiratory frequency, Ppeak; peak airway pressure, Cdyn; dynamic compliance

The anesthesia course is shown in (Fig. 1). In the operating room, the patient’s back was disinfected with 1.0% chlorhexidine gluconate. Local anesthesia was administered using 1.0% lidocaine with adrenaline, and an epidural catheter was inserted. Induction of anesthesia was　initiated after vital signs were confirmed stable.

Following administration of 10 mg remimazolam and initiation of a 60 mg/h infusion with remifentanil 0.2 µg/kg/min, the patient rapidly developed tachycardia and coughing. As ventilation became increasingly difficult, 50 mg of rocuronium was administered, and a 37-Fr double-lumen endotracheal tube was placed.

**Fig. 2:**
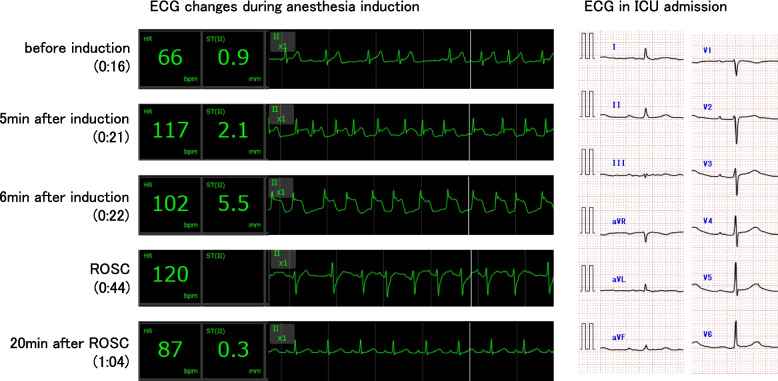
Serial electrocardiographic changes. The time points in this figure correspond to the clinical events in Fig. 1. Note the immediate ST-segment elevation following remimazolam administration

After intubation, an ECG revealed marked ST-segment elevation (Fig. 2). Systolic blood pressure dropped to 40 mmHg, and airway pressure increased (Fig. 1). KS-associated anaphylactic shock was suspected. Despite immediate intravenous adrenaline administration and rapid fluid resuscitation, the patient progressed to pulseless electrical activity. Cardiopulmonary resuscitation was initiated; however, effective perfusion pressure could not be maintained with only chest compressions, ECPR was then started. Return of spontaneous circulation (ROSC) occurred 15 min after cardiac arrest with venoarterial extracorporeal membrane oxygenation (VA-ECMO) established 2 min later. In total, 6 mg of adrenaline was administered intravenously.

A post-ROSC ECG showed rapid resolution of ST changes (Fig. 2). Echocardiography revealed diffuse, severe left ventricular dysfunction without regional wall motion abnormalities. Continuous nicorandil infusion at 2 mg/h was initiated to relieve coronary vasospasm. Following a discussion with the cardiologists, transient coronary vasospasm was suspected based on the clinical course. Stabilization was prioritized and emergent coronary angiography was deferred; the patient was admitted to the intensive care unit (ICU). Post-admission, 12-lead ECG showed no significant ST-segment alterations (Fig. 2), and cardiac enzyme levels were slightly elevated [creatine kinase (CK): 99 IU/L; CK–myocardial band: 47 IU/L]. Consequently, a decision to postpone the assessment of the coronary arteries until a later date was made. Coronary computed tomography (CT) performed 6 weeks later confirmed no major stenosis, consistent with type 1 KS.

**Fig. 3:**
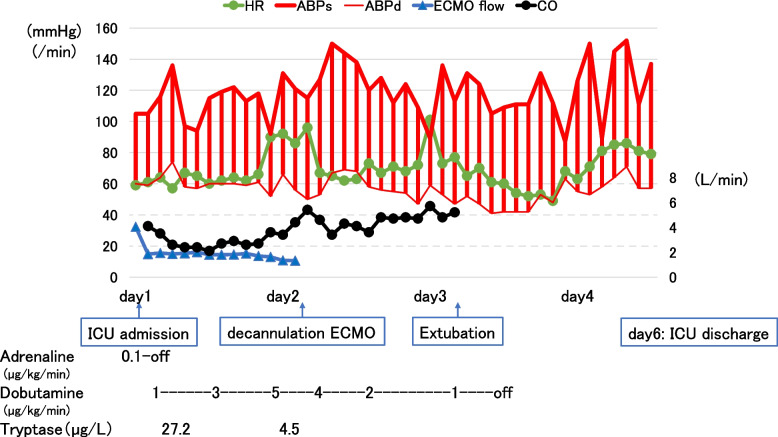
Clinical course in intensive care unit. Cardiac output gradually recovered during the stabilization period. HR; heart rate, ABPs; systolic arterial blood pressure, ABPd; diastolic arterial blood pressure, ECMO; extracorporeal membrane oxygenation, CO: cardiac output

Serum tryptase levels were 49.5 µg/L at 45 min and 27.2 µg/L at 5 h post onset; the reference level at 24-h post onset is 4.5 µg/L, suggesting immunoglobulin E (IgE)-mediated anaphylactic shock. The ICU clinical course is shown in (Fig. 3). VA-ECMO was decannulated using dobutamine on day 2. Ventricular function in echocardiography and cardiac output recovered steadily and inotropes could be stopped on day 3. The patient was then extubated and discharged on day 18 without neurological sequelae.

**Fig. 4:**
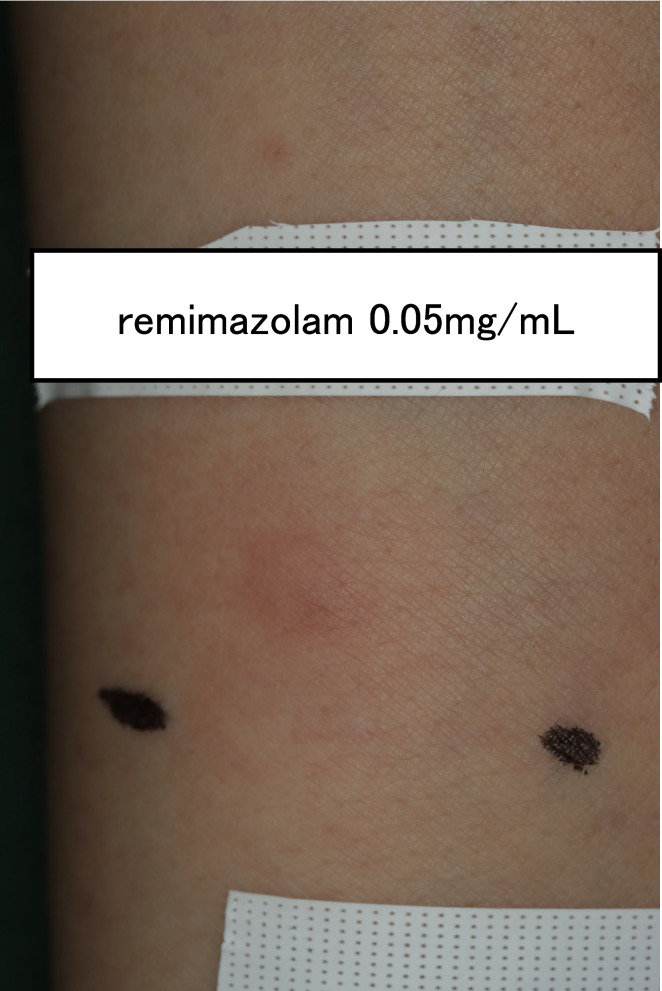
Photograph of the intradermal test

Nine weeks after the anaphylactic episode, skin testing was performed to identify the causative agent. Suspected drugs included all drugs used in anesthesia induction: adrenaline-containing 1.0% lidocaine, 1.0% chlorhexidine gluconate, remimazolam, remifentanil, and rocuronium. Midazolam was not included because it had a history of safe use; however, as will be discussed subsequently, trials may have been necessary. All drugs were negative on skin prick testing, but intradermal testing showed a positive reaction only to remimazolam (0.05 mg/mL; Fig. 4). Based on these results, remimazolam-induced anaphylaxis was suspected.

## Discussion

In this case report, remimazolam-induced anaphylaxis was possibly complicated by type 1 KS, resulting in refractory cardiac arrest. Early initiation of VA-ECMO was effective, and the patient survived with a favorable neurological outcome.

In this case report, the clinical course strongly suggested KS type 1, although coronary angiography was not performed and there was no direct evidence of vasospasm. The normal coronary artery seen on the coronary CT; with the ST-segment changes that preceded circulatory collapse and resolved quickly support the diagnosis. KS arises from mast cell and platelet activation caused by the inflammatory mediators such as histamine, platelet-activating factor, arachidonic acid products, and a variety of cytokines and chemokines. It is classified into three types: type 1 involves coronary spasm induced by inflammatory mediators, type 2 is associated with coronary plaque rupture, and type 3 involves coronary stent thrombosis [1]. Abdelghany et al. reported type 1 as the most common variant (72.6% of cases) [2]. Various drugs can trigger Perioperative KS, including anesthetics [4]. No clear reports exist of remimazolam-induced KS; however, previous cases of remimazolam-induced anaphylaxis noted ST elevation [5].

Perioperative KS develops rapidly, often presenting severe symptoms that require mechanical circulatory support [4]. The reported mortality rate for KS is ~ 7.0% [6], higher than the 1.4%–4.8% rate observed for perioperative anaphylaxis [7], underscoring its seriousness. In the presented case, KS occurred immediately after remimazolam administration, with a rapid ST elevation, prolonged cardiac arrest, and the need for mechanical circulatory support. Regarding adrenaline administration, some guidelines caution that it may worsen coronary spasm or ischemia [8]. In contrast, adrenaline is a specific treatment for anaphylaxis; in addition to improving shock through its inotropic and vasoconstrictive effects, adrenaline inhibits the release of chemical mediators from inflammatory cells [9]. These effects improve the underlying pathophysiology of anaphylaxis itself. Caution should be exercised when using adrenaline in cases of KS. It should be considered in cases of refractory hypotension or cardiac arrest [10]. Thus, clinicians should consider the possibility of KS when managing remimazolam-induced anaphylaxis.

Refractory anaphylaxis is commonly defined as a failure to respond to repeated adrenaline doses [11]. Treatment options include noradrenaline, vasopressin, or methylene blue. In cases of persistent cardiac arrest, ECPR should be considered [11, 12]. The 2025 American Heart Association guidelines also recommend ECPR for anaphylaxis when standard resuscitation is ineffective [3]. In this case report, refractory anaphylaxis was attributed to both KS and concomitant severe bronchospasm. Bronchospasm increases intrathoracic pressure, leading to decreased venous return. Furthermore, increased pulmonary vascular resistance reduces right ventricular output, resulting in delayed systemic perfusion and a delayed clinical onset of adrenaline. Therefore, ECPR should be considered for anaphylaxis complicated by KS or bronchospasm when resuscitation is difficult. ECMO requires time for initiation; thus, early decision-making is essential. In the current case, ECMO was established simultaneously with ROSC. However, severe cardiac dysfunction persisted post-ROSC, and ECMO provided effective circulatory support until cardiac function recovered. Persistent cardiac dysfunction was caused by several factors in this case report, including stunning after defibrillation, myocardial ischemia due to KS, and prolonged circulatory collapse. Thus, for refractory anaphylaxis, ECMO is beneficial during resuscitation and posttreatment, and its early initiation should be considered when available.

In this case report, remimazolam was suspected as the causative agent via skin testing. The patient had previously received multiple midazolam administrations without adverse reactions. Cross-reactivity between remimazolam and midazolam is documented [13], suggesting that sensitization may have occurred after repeated midazolam exposures. There are some limitations to confirming the causative agent. Dextran 40, an additive in remimazolam formulations, can cause anaphylaxis, typically via non-IgE–mediated mechanisms, and skin testing is unreliable for detection [14]. In the reported case, a positive skin test indicated IgE-mediated reaction, and a lower likelihood of dextran 40 association. To elucidate the mechanism underlying remimazolam-induced anaphylaxis, testing for midazolam and dextran 40 should be considered when feasible.

In summary, the patient in this case report developed remimazolam-induced anaphylaxis and a suspected KS type 1 complication. Early initiation of ECPR was effective in managing refractory cardiac arrest, allowing full recovery without neurological sequelae.

## Data Availability

Data relevant to this case report are not publicly available because of concerns regarding patient privacy but are available from the corresponding author upon reasonable request.

## Abbreviations

CK: Creatine kinase
ECG: Electrocardiogram
ECPR: Extracorporeal cardiopulmonary resuscitation
KS: Kounis syndrome
ROSC: Return of spontaneous circulation
VA-ECMO: Venoarterial extracorporeal membrane oxygenation
ICU: Intensive care unit
IgE: Immunoglobulin E
